# Production of IL-6 and Phagocytosis Are the Most Resilient Immune Functions in Metabolically Compromised Human Monocytes

**DOI:** 10.3389/fimmu.2021.730672

**Published:** 2021-10-19

**Authors:** Pierre-Louis Krauss, Moritz Pfeiffenberger, Alexandra Damerau, Thomas Buttgereit, Yuling Chen, Timo Gaber, Frank Buttgereit

**Affiliations:** ^1^ Department of Rheumatology and Clinical Immunology, Charité — Universitätsmedizin Berlin, corporate member of Freie Universität Berlin and Humboldt-Universität zu Berlin, Berlin, Germany; ^2^ German Rheumatism Research Centre (DRFZ) Berlin, a Leibniz Institute, Berlin, Germany; ^3^ Department of Dermatology, Venerology, and Allergology, Charité — Universitätsmedizin Berlin, corporate member of Freie Universität Berlin and Humboldt-Universität zu Berlin, Berlin, Germany

**Keywords:** immunometabolism, bioenergetics, IL-6, phagocytosis, human monocytes, energy, ATP, lack of glucose availability

## Abstract

At sites of inflammation, monocytes carry out specific immune functions while facing challenging metabolic restrictions. Here, we investigated the potential of human monocytes to adapt to conditions of gradually inhibited oxidative phosphorylation (OXPHOS) under glucose free conditions. We used myxothiazol, an inhibitor of mitochondrial respiration, to adjust two different levels of decreased mitochondrial ATP production. At these levels, and compared to uninhibited OXPHOS, we assessed phagocytosis, production of reactive oxygen species (ROS) through NADPH oxidase (NOX), expression of surface activation markers CD16, CD80, CD11b, HLA-DR, and production of the inflammatory cytokines IL-1β, IL-6 and TNF-α in human monocytes. We found phagocytosis and the production of IL-6 to be least sensitive to metabolic restrictions while surface expression of CD11b, HLA-DR, production of TNF-α, IL-1β and production of ROS through NOX were most compromised by inhibition of OXPHOS in the absence of glucose. Our data demonstrate a short-term hierarchy of immune functions in human monocytes, which represents novel knowledge potentially leading to the development of new therapeutics in monocyte-mediated inflammatory diseases.

## Introduction

Human monocytes use energy, mostly in the form of ATP, for housekeeping functions such as cation transport and the generation of macromolecules as well as for a variety of specific tasks. These tasks include transendothelial migration, phagocytosis (1, 2), presentation of antigens (3), differentiation into macrophages, dendritic cells (4) and osteoclasts (5), synthesis and secretion of cytokines such as interleukin-1β (IL-1β), IL-6 and tumor necrosis factor (TNF-alpha) (2), and the production of reactive oxygen species (ROS) (6). The correct execution of these tasks necessitates sufficient supply of energy and intermediates. Without energy, the function of these important immune cells would certainly fail (7). In addition, low availability or even the absence of glucose carbon will compromise a series of metabolic pathways (such as the pentose phosphate pathway, the serine synthesis pathway, and the glycerol phosphate shuttle), which results in a reduced synthesis of intermediates leading to a potentially negative influence on cellular activation (8–12). Against this background, it should be noted that monocytes face variable and demanding microenvironmental conditions (13) when migrating from the blood into multiple tissues. Because of high metabolic activity, the “battlefields” of monocytes (e.g., sites of acute and chronic tissue inflammation such as wound healing, inflamed joints, sites of ischemia and in growing tumors) are usually characterized by diverse and often extremely compromised conditions. These include very low supply of oxygen, glucose and other nutrients, low pH, and increased lactate levels – which collectively lead to less effective synthesis of ATP and intermediates both being critical for cellular activation, ultimately causing limited availability of energy and building blocks (12, 14, 15). Therefore, immune cells such as human monocytes use different metabolic programs to meet their cellular energy needs and for the generation of biomolecules, which enable them to cope with these challenging, metabolically restricted conditions (8–12). This also means that they must be metabolically highly dynamic, and that they need to prioritize their functions when energy and substrate supply is limited. In detail, cellular adaptations in energy and intermediate metabolism affect the immune response both qualitatively and quantitatively (7, 8, 13, 16–18). However, the exact quantitative consequences of such compromised conditions on the functions of monocytes are still elusive.

In this study, we therefore hypothesized metabolically compromised conditions to affect the functions of monocytes in a differential (hierarchical) manner. Thus, monocytes will reweigh or switch off their specific tasks such as ROS production, expression of surface markers, cytokine synthesis and phagocytosis one after another (depending on its importance) when subjected to compromised conditions. To test this hypothesis, we modeled metabolic restrictions by gradually reducing the mitochondrial ATP production of human monocytes under conditions of glucose deprivation in order to assess crucial immune functions at pathophysiological conditions.

## Material and Methods

### Antibodies and Reagents

For cell stimulation and Golgi transport block, lipopolysaccharide (LPS) and Brefeldin A (BFA) were purchased from Sigma-Aldrich (St. Louis, USA). For the analysis of oxygen consumption, Myxothiazol (MYX), Oligomycin A, and the pan- NADPH oxidase (NOX) inhibitor VAS-2870 were purchased from Sigma-Aldrich (St. Louis, USA). Flebogamma (highly purified, unmodified, human IgG: IgG1 66.6%, IgG2 28.5%, IgG3 2.7%, IgG4 2.2%) was purchased from Grifols (Frankfurt, Germany). For intracellular ROS (iROS) detection, 5-(and 6-) chloromethyl-2’,7’-dichlorodihydrofluorescein diacetate, acetyl ester (CM-H_2_DCFDA) was purchased from Invitrogen GmbH (Karlsruhe, Germany). For assessments of phagocytosis, FITC labeled *E. coli* were applied using the Phagotest™ (Glycotope, Berlin, Germany). For flow cytometry, Fc receptor block was achieved by adding Flebogamma (highly purified, unmodified, human IgG: IgG1 66.6%, IgG2 28.5%, IgG3 2.7%, IgG4 2.2%) purchased from Grifols (Frankfurt, Germany). All antibodies used are listed in **Table 1**. Following the European guidelines for flow cytometry, isotype controls were exclusively used in the establishment phase of staining protocol to verify effectiveness of Fc blocking by Flebogamma, since isotype controls do not control staining specificity (19).

**Table 1:** Antibodies used for flow cytometry.

Antibody	Conjugate	Manufacturer	Catalog Number	Species of origin	Dilution
**anti-CD14**	APC-Cy7	Biolegend	301820	mouse IgG2a, κ	1:25
**anti-CD14**	PE-Vio770	Miltenyi	130-110-521	REA599, recombinant	1:50
**anti-CD16**	PE-Vio770	Miltenyi	130-113-394	REA423, recombinant	1:50
**anti-CD16**	VioBlue	Miltenyi	130-099-080	mouse IgMκ	1:11
**anti-CD80**	APC	Miltenyi	130-097-204	mouse IgG1κ	1:11
**anti-CD11b**	PE	DRFZ	n/a	mouse IgG2b	1:100
**anti-HLA-DR**	VioBlue	Miltenyi	130-113-406	mouse IgG2aκ	1:11
**anti-hTNF-α**	APC-Vio770	Miltenyi	130-120-491	human IgG1	1:50
**anti-hIL-1β**	PE	Thermofisher	MA5-23546	mouse/IgG1	1:10
**anti-hIL-6**	APC	Miltenyi	130-096-088	rat IgG1κ	1:11

### Preparation of PBMC Isolation of CD14+ Monocytes, and Cell Culture

Monocytes were isolated from heparinized peripheral blood of healthy volunteers after giving written informed consent (ethical approval EA1/207/17 Charité - Universitätsmedizin Berlin). In brief, peripheral blood mononuclear cells (PBMC) from heparinized peripheral blood were isolated by density gradient centrifugation using Ficoll-Paque™ Plus (GE Healthcare, Chicago, USA). CD14^+^ monocytes were enriched from PBMC with >95% purity and >95% viability (data not shown, gating strategy provided in **Figure S1**) by using anti-human CD14 conjugated magnetic beads (Miltenyi Biotec, Bergisch Gladbach, Germany) and then immediately used for the experiments. Monocytes were cultured at 37°C in a humidified and atmosphere with 5% CO_2_ (Binder, Tuttlingen, Germany) in aliquots of 300 µL at a density of 1x10^7^ cells/ml in 13 mL round bottom polypropylene tubes (Sarstedt, Nümbrecht, Germany) under orbital shaking at 120 min^-1^ (KS250basic, IKA-Labortechnik, Staufen, Germany) in glucose free RPMI 1640 (Thermofisher, Waltham, USA) supplemented with 10% (v/v) dialyzed (glucose-free) human AB serum (Sigma-Aldrich, St. Louis, USA).

### Quantification of Oxygen Consumption

For the measurement of the oxygen consumption rate (OCR) following treatment with MYX, VAS2870, and oligomycin A (all from Sigma-Aldrich, St-Louis, USA), CD14+ monocytes were resuspended in glucose free RPMI-1640 [permits oxidative phosphorylation (OXPHOS), but not glycolysis] supplemented with 10% (v/v) dialyzed (glucose-free) human AB serum (Sigma-Aldrich, St. Louis, USA). The cells were pre-treated 2 hours with 100 ng/mL LPS derived from *E. coli* (Sigma-Aldrich, St. Louis, USA) or left untreated in the absence (vehicle control: 1% Dimethylsulfoxide, DMSO) or presence of 2 pmol MYX/10^6^ cells (MYX1) or 4 pmol MYX/10^6^ cells (MYX2), 50 µM VAS2870, 1 µM oligomycin A and 20 µM Carbonylcyanid-p-trifluoromethoxyphenylhydrazon (FCCP) (Sigma-Aldrich, St. Louis, USA), respectively. MYX irreversibly inhibits complex III of the mitochondrial respiratory chain, reducing mitochondrial biosynthesis of ATP (20). OCR was measured amperometrically in 100 µl of cell suspension (3 – 9.0 x 10^6^ cells/ml) with a SI130 microcathode Clark-type oxygen electrode and Mitocell MT200 respirometry system (Strathkelvin, Scotland, UK). After an incubation of 2 and 6 hours, basal OCR of MYX-treated cells was assessed, followed by treatment with VAS2870 and oligomycin A. OCR committed to the NADPH-Oxidase (ΔOCRV), OCR committed to the mitochondrial ATP production (ΔOCRO), VAS2870/oligomycin A insensitive OCR and mitochondrial reserve capacity were obtained by following a strictly timed protocol (shown in **Figure S2**)

### Assessment of Cell Viability

Apoptosis and necrosis of monocytes were quantified by Annexin V (Biolegend) and 7-AAD staining (BD Biosciences, San Jose, USA) (gating strategy provided in **Figure S1**, reagent concentration provided in **Table 2**) according to the manufacturer’s instructions. Data were acquired using a BD FACSCanto™ II (BD Biosciences, San Jose, USA) and processed by FlowJo v7.6.5 (BD Biosciences, San Jose, USA).

**Table 2:** Staining compounds used for flow cytometry.

Staining compound	Manufacturer	Catalog Number	Dilution
*7AAD*	*BD Pharmingen*	*559925*	*1:20*
*Annexin-V-PE*	*Biolegend*	*640908*	*1:200*
*Annexin-V-FITC*	*Biolegend*	*640906*	*1:400*
*Zombie Green*	*Biolegend*	*423111*	*1:400*

### Assessment of Fuel Oxidation

Seahorse XF Mito Fuel Flex Test was performed on XFe96 Bioanalyzer (Agilent Technology). All assays were performed following manufacture’s protocols. In brief, monocytes were cultured in a 96-well assay plate in RPMI supplemented with 10% (v/v) dialyzed, human AB serum at 10^5^ cells per well under glucose-free conditions. Cells were stimulated for 4 h with both 100 ng/mL LPS and 2 pmol MYX/10^6^ cells (MYX1) or 4 pmol MYX/10^6^ cells (MYX2) or LPS alone. The Mito Fuel Flex Test inhibits the import of three metabolic substrates (pyruvate, fatty acids and glutamine) with mitochondrial pyruvate carrier inhibitor UK5099 (2 µM), carnitine palmitoyltransferase 1A inhibitor etomoxir (4 µM), or glutaminase inhibitor BPTES (3 µM). Mitochondrial stress and glycolytic parameters were measured *via* OCR in pmol/min/1x10^5^ cells. Metabolic parameters were calculated according to the manufacturer’s instructions (Agilent Technology). Thus, cellular dependence on each of metabolite to fuel mitochondrial metabolism can be analyzed.

### Quantification of ROS Production by Flow Cytometry

To measure cellular oxidative stress, iROS were detected using 5 (and 6) - chloromethyl-2’,7’-dichlorodihydrofluorescein diacetate, acetyl ester (CM-H_2_DCFDA; Invitrogen GmbH, Karlsruhe, Germany). Cells were incubated 30 min in PBS (DRFZ, Berlin, Germany) with 5 µM CM-H_2_DCFDA, washed with glucose-free RPMI-1640 and incubated 2 hours in glucose-free RPMI-1640 and 10% (v/v) dialyzed human AB serum, with or without 100 ng/mL LPS and with 1% DMSO, MYX1 or MYX2. After incubation, cells were washed with PBS and subsequently stained with anti-human CD14-APC-Cy7 (Biolegend, San Diego, USA). To exclude dead and apoptotic cells, cells were stained with Annexin V-PE (Biolegend, San Diego, USA) and 7-AAD (BD Biosciences, San Jose, USA) (gating strategy provided in **Figure S3**, reagent concentration provided in **Tables 1**, **2**). Data were acquired using a BD FACSCanto™ II (BD Biosciences, San Jose, USA) and processed by FlowJo v7.6.5 (BD Biosciences, San Jose, USA).

### Quantification of Phagocytosis

Phagocytosis was quantified using the Phagotest™ (Glycotope, Berlin, Germany) according to the manufacturer’s instructions. Data were acquired using a BD FACSCanto™ II (BD Biosciences, San Jose, USA) and processed by FlowJo v7.6.5 (BD Biosciences, San Jose, USA).

### Quantification of Cellular ATP

ATP content of monocytes was assessed with the CLS II KIT (Roche, Mannheim, Germany) according to the manufacturer’s instructions. Luminescence of all samples was quantified using the SynergyHT plate-reader (BioTek, Bad Friedrichshall, Germany).

### Quantification of Cytokine Production

For the intracellular measurement of TNF-α, IL-1β, and IL-6, human monocytes were pre-stimulated for 1 hour with 100 ng/mL LPS in glucose-free RPMI with 10% (v/v) dialyzed human AB serum, with or without 100 ng/mL LPS and with 1% DMSO, MYX1 or MYX2. Protein transport was blocked by adding Brefeldin A (Sigma-Aldrich, St-Louis, USA) at a concentration of 10 µg/mL for another 1 or 3 hours to achieve intracellular cytokine accumulation. Cells were stained using Zombie Green (Biolegend, San Diego, USA) according to the manufacturer’s instructions. Subsequently, cells were stained protected from light using the IC staining Kit (Miltenyi Biotech, Bergisch Gladbach, Germany) according to manufacturer´s instructions. Intracellular cytokines were stained by using antibodies against hTNF-α, hIL-1β, hIL-6 and as described above (**Table 1**). Data were acquired using a BD FACSCanto™ II (BD Biosciences, San Jose, USA) and processed by FlowJo v7.6.5 (BD Biosciences, San Jose, USA).

### Quantification of Cytokine Secretion

Monocytes were cultured in a 96-well plate in glucose-free RPMI supplemented with 10% (v/v) dialyzed, human AB serum at 10^5^ cells per well. Cells were stimulated for 6 h with both 100 ng/mL LPS and 2 pmol MYX/10^6^ cells (MYX1) or 4 pmol MYX/10^6^ cells (MYX2) or LPS alone. Subsequently, supernatants were collected and TNF-α, IL-1β, IL-6 and IL-6/IL-6 Rα complex release was measured by ELISA (R&D Systems, Minneapolis, USA) following the manufacturer’s instructions.

### Quantification of Surface Marker Expression

After blocking the unspecific binding of Fc-receptor for 10 min on ice in 10 µL of a solution containing 5 mg/ml human IgG (IgG1 66.6%, IgG2 28.5%, IgG3 2.7%, IgG4 2.2%; Flebogamma, Grifols, Frankfurt, Germany), cells were washed in PBS/BSA (DRFZ, Berlin, Germany). Antibody staining was performed for 10 min at 4°C for the detection of surface marker expression using anti-CD14-FITC and anti-CD11b-PE (both from DRFZ, Berlin, Germany), anti-CD16-PE-Vio770, anti-CD80-APC and anti-HLA-DR-VioBlue (all from Miltenyi Biotech, Bergisch Gladbach, Germany; see also **Table 1**). Apoptosis and necrosis of monocytes were quantified by Annexin V (Biolegend, San Diego, USA) and 7-AAD staining (BD Biosciences, San Jose, USA) (gating strategy provided in **Figure S1**, reagent concentration provided in **Tables 1**, **2**) according to the manufacturer’s instructions. Data were acquired using a BD FACSCanto™ II (BD Biosciences, San Jose, USA) and processed by FlowJo v7.6.5 (BD Biosciences, San Jose, USA).

### Quantification of Gene Expression

Monocytes were cultured in a 96-well plate in glucose-free RPMI supplemented with 10% (v/v) dialyzed, human AB serum at 10^5^ cells per well. Cells were stimulated for 6 h with both 100 ng/mL LPS and 2 pmol MYX/10^6^ cells (MYX1) or 4 pmol MYX/10^6^ cells (MYX2) or LPS alone. Subsequently, cell pellets were collected and total RNA was extracted using the RNA Isolation Kit: RNeasy Mini Kit (QIAGEN, Hilden, Germany) according to the manufacturer’s instructions. cDNAs were synthesized by reverse transcription using the TaqMan™ Reverse Transcription Kit (ThermoScientific, Waltham, USA) according to the manufacturer’s instructions. After transcription, cDNAs were stored at -20°C until further processing. Quantification of gene expression was performed by qPCR using the DyNAmo Flash SYBR Green qPCR Kit (Thermo Fisher, Waltham, USA) according to the manufacturer’s instructions and assessed in a Stratagene Mx3000P (Agilent Technologies, California, USA) using the following program: initial denaturation, 7 min at 95°C; amplification, 60 cycles with 10 s at 95°C, 7 s at 60°C and 9 s at 72°C. Melting curve analysis was assessed by a stepwise temperature increase from 50°C to 95°C every 30 s. Data were normalized to the expression of elongation-factor 1-α (*EF1A*) and to the respective control using the ΔΔCt-method. All primers were purchased from TIB Molbiol (Berlin, Germany) and are listed in **Table 3**.

**Table 3:** Primer used for quantitative PCR.

Gene symbol	Gene name	Forward primer	Reverse primer
*EF1A*	Elongation Factor 1-alpha	*GTTGATATGGTTCCTGGCAAGC*	*TTGCCAGCTCCAGCAGCCT*
*IL1B*	Interleukin 1-β	*AGCTACGAATCTCCGACCAC*	*CGTTATCCCATGTGTCGAAGAA*
*TNFA*	Tumor Necrosis Factor-α	*GTCTCCTACCAGACCAAG*	*CAAAGTAGACCCTGCCCAGACTC*
*IL6*	Interleukin 6	*TACCCCCAGGAGAAGATTCC*	*TTTTCTGCCAGTGCCTCTTT*
*IL6RA*	Interleukin 6 Receptor	*CCCCTCAGCAATGTTGTTTGT*	*CTCCGGGACTGCTAACTGG*
*HK1*	Hexokinase 1	*CACATGGAGTCCGAGGTTTATG*	*CGTGAATCCCACAGGTAACTTC*
*CPT1*	Carnitine palmitoyl transferase I	*ATCAATCGGACTCTGGAAACGG*	*TCAGGGAGTAGCGCATGGT*
*SDHA*	Succinate Dehydrogenase Complex Flavoprotein Subunit A	*CAGCATGTGTTACCAAGCTGT*	*GGTGTCGTAGAAATGCCACCT*
*PDHA1*	Pyruvate dehydrogenase E1 component subunit alpha	*ATGGAATGGGAACGTCTGTTG*	*CCTCTCGGACGCACAGGATA*

### Statistical Analysis

Data are depicted as mean ± SEM. Differences between independent groups were verified using the non-parametric Mann-Whitney U test. Differences between dependent samples were verified using the non-parametric Wilcoxon signed rank test. The probability values of p<0.05 were considered statistically significant.

## Results

### Human Monocytes Survive Strong Inhibition of Mitochondrial Respiration in the Absence of Glucose

**Figure 1:**
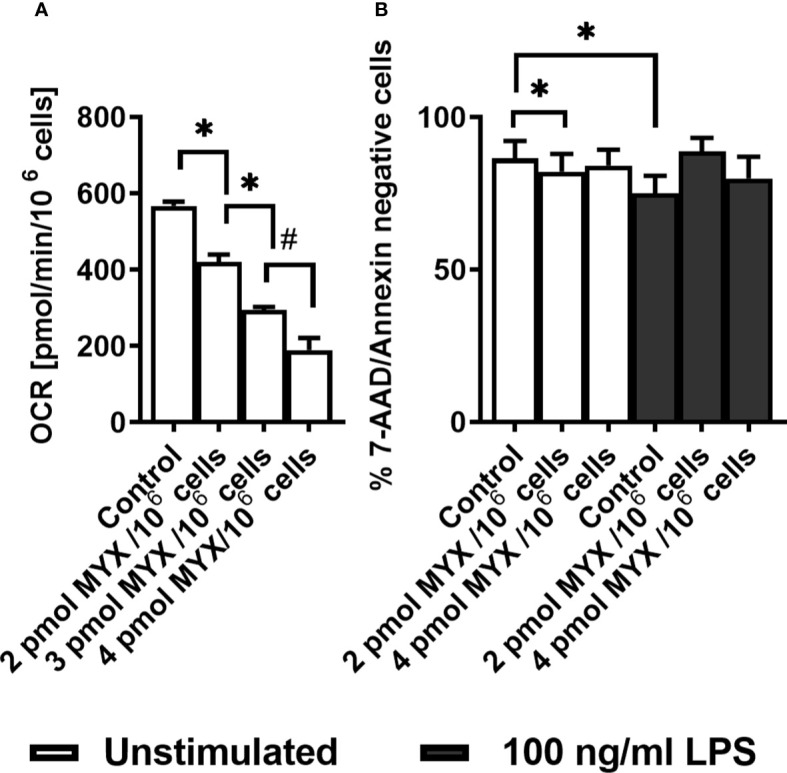
Human monocytes survive strong metabolic restriction. **(A)** Titration of Myxothiazol (MYX) in medium without glucose in the presence of MYX (pmol/10^6^ cells) or 1% (v/v) DMSO (vehicle control) under glucose free conditions (n = 3-6). Values are expressed as mean ± SEM (*p < 0.05 Wilcoxon signed rank test, ^#^p < 0.05 Mann-Whitney U Test). **(B)** Viability of quiescent and stimulated (100 ng/mL LPS) human monocytes after 6 h of incubation in the presence or absence of MYX (pmol/10^6^ cells) or vehicle control (1% (v/v) DMSO) under glucose free conditions (n = 6). Values are expressed as mean ± SEM (*p < 0.05 Wilcoxon signed rank test).

The basal OCR of quiescent human monocytes after 2 hours of preincubation under glucose free conditions was 566 ± 11.63 pmol/min/10^6^ cells. Treatment with increasing quantities of MYX significantly reduced the basal OCR to 419.7 ± 47.71 (2 pmol MYX/10^6^ cells), 293.0 ± 19.81 (3 pmol/10^6^ cells) and 189.3 ± 54.52 pmol/min/10^6^ cells (4 pmol MYX/10^6^ cells), which corresponded to an inhibition of roughly 25, 50 and 66% of the basal oxygen consumption rate (**Figure 1A**). After 6 hours of incubation under glucose free conditions, with or without LPS and/or MYX, we observed minor differences in the rates of viable cells: compared to the untreated control, 2 pmol MYX/10^6^ cells reduced the viability rate of quiescent monocytes from 86.38 ± 5.79 to 82.12 ± 5.82 (p=0.0313), and treatment with LPS significantly reduced the viability rate of the monocytes to 75.02 ± 5.74 (p=0.0156), respectively. The combination of both, treatment with 2 and 4 pmol MYX/10^6^ cells and stimulation with LPS, did not affect the viability of human monocytes (**Figure 1B**). We therefore used the MYX doses of 2 and 4 pmol/10^6^ cells (named MYX1 and MYX2 respectively) for a maximum incubation duration of 6 hours in the subsequent experiments.

### Metabolic Restriction Alters ROS and ATP Production in Human Monocytes

**Figure 2:**
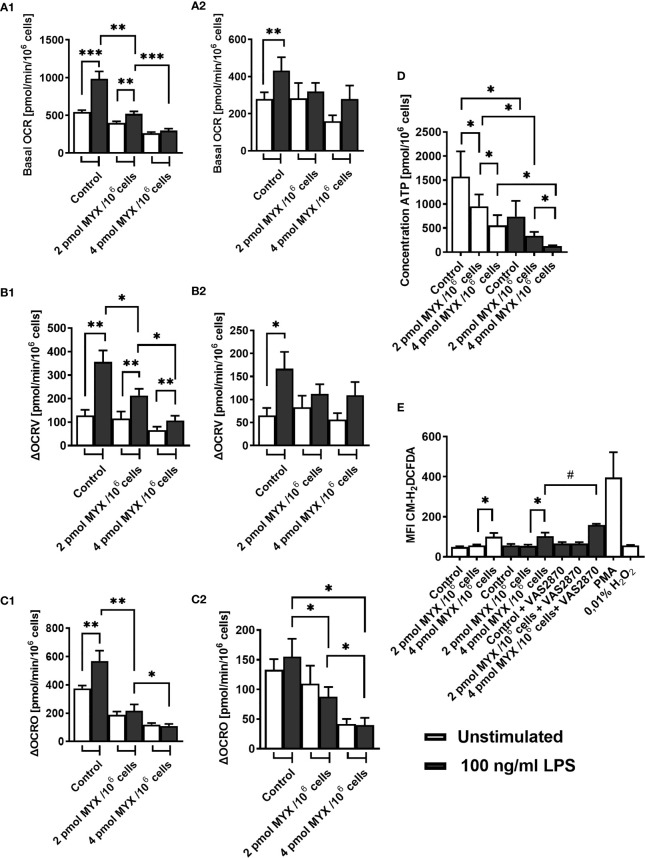
Metabolic restriction with MYX alters ROS and ATP production in human monocytes in the absence of glucose. **(A–C)** Oxygen consumption rate (OCR) and inhibitor sensitive oxygen consumption rate (ΔOCR) of quiescent and stimulated (100 ng/mL LPS) human monocytes after 2 h (A1-C1) 6 h (A2-C2) of incubation in the presence or absence of Myxothiazol (MYX) (pmol/10^6^ cells) or 1% (v/v) DMSO (vehicle control) under glucose free conditions. After preincubation, **(A)** basal OCR, **(B)** VAS2870 sensitive OCR (ΔOCRV) and **(C)** oligomycin sensitive OCR (ΔOCRO) were assessed (mean ± SEM; n = 9 – 10). **(D)** Intracellular ATP content of quiescent and stimulated (100 ng/mL LPS) human monocytes after 3 h of incubation in the presence or absence of MYX (pmol/10^6^ cells) or 1% (v/v) DMSO (vehicle control) under glucose free conditions was measured by bioluminescence (mean ± SEM; n = 6). **(E)** Median fluorescence intensity of quiescent and stimulated (100 ng/mL LPS) human monocytes after 2 h of incubation in the presence or absence of MYX (pmol/10^6^ cells) or 1% (v/v) DMSO (vehicle control) and/or 50 µM VAS2870, or 100ng/ml PMA or H_2_O_2_ under glucose free conditions, stained with CM-H_2_DCFDA (mean ± SEM; n = 3 – 5); *p < 0.05, **p < 0.01, ***p < 0.001. Wilcoxon signed rank test. ^#^p < 0.05; Mann-Whitney U Test.

Next, we investigated the OCR of human monocytes following treatment with LPS and/or MYX1 and MYX2. Since the cells showed high viability within the period of 6 hours, we chose to assess the basal OCR and the partial OCR contributing to the production of ROS through the NADPH oxidase (ΔOCRV, **Figure S1**) and mitochondrial production of ATP (ΔOCRO, **Figure S1**) after 2 and 6 hours of incubation under glucose free conditions. After 2 hours, stimulation with LPS alone significantly increased the mean basal OCR by 81.55% (p=0.001) (**Figure 2A**). MYX1 diminished this effect to an increase of 29.50% (p=0.0098). MYX2 abrogated the stimulating effect of LPS on the basal OCR. OCR of LPS-stimulated monocytes differed from each other (p=0.002 and p=0.001): with an increasing dose of MYX, we observed a stronger decrease in the basal OCR, indicating a dose response relationship (**Figure 2A**). We observed a similar dose response relationship in the ΔOCRV: the ΔOCRV of monocytes stimulated with LPS decreased with higher quantities of MYX (p=0.0195 and p=0.0186). Stimulation with LPS alone increased the mean ΔOCRV by 178.1% (p=0.0029). Upon MYX1 treatment, stimulation with LPS increased the mean ΔOCRV by 85.22%(p=0.0029). However, MYX2-treatment reduced this to 60.61% (p=0.0186) (**Figure 2B**). In the case of mean ΔOCRO, LPS-stimulated consumption rates were also differed from each other (p=0.002 and p=0.0322), and stimulation with LPS alone induced an increase by 51.47% (p=0.001). However, LPS did not induce any additional ΔOCRO upon MYX1 or MYX2 treatment. (**Figure 2C**). After 6 hours of incubation under the same conditions, stimulation with LPS alone induced an increase only in mean basal OCR (by 55.40%, p=0.0078) and in ΔOCRV (by 154.5%, p=0.0195). ΔOCRO of stimulated monocytes showed a dose dependent decrease, but the stimulation itself did not induce any additional ΔOCRO (**Figures 2A–C**). Additionally, stimulation with LPS decreased the mitochondrial reserve capacity of the monocytes at all metabolic levels investigated, with no difference between control, MYX1 and MYX2 (data not shown). However, unstimulated cells showed a decrease of mitochondrial reserve capacity compared to the control only upon MYX2-treatment. Treatment with LPS or MYX had no significant impact on NOX or ATPase independent OCR (data not shown).

**Figure 3:**
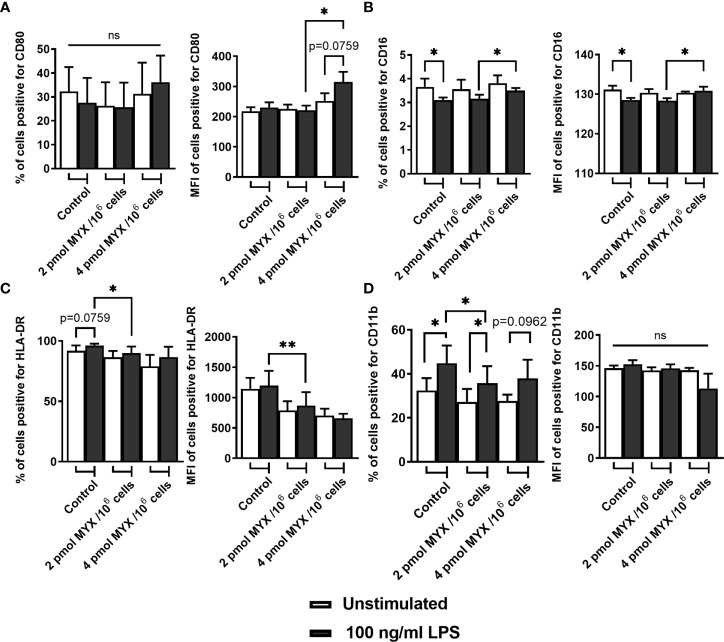
Metabolic restriction with MYX modifies expression of surface markers of activation. **(A-D)** Percentage and median fluorescence intensity of quiescent and stimulated (100 ng/mL LPS) human monocytes positive for staining of surface **(A)** CD80, **(B)** CD16, **(C)** HLA-DR and **(D)** CD11b in the presence or absence of MYX (pmol/10^6^ cells) or vehicle control (1% (v/v) DMSO) under glucose free conditions. Cells were incubated for 4 hours. (mean ± SEM; n = 6; *p < 0.05; **p < 0.01; Wilcoxon signed rank test). ns, not significant.

In addition to ΔOCRO and ΔOCRV, we analyzed cellular ATP and iROS levels of primary human monocytes (**Figures 2D, E**). The cellular ATP content of the monocytes decreased following stimulation with LPS (p=0.0156). However, LPS combined with MYX treatment reduced the ATP content compared to unstimulated controls (both p=0.0156), and there was also a difference between MYX1 and MYX2 in the stimulated group (p=0.0313) ATP concentration of LPS stimulated cells upon MYX2 treatment was roughly 8% compared to the unstimulated control (**Figure 2D**). LPS stimulation did not increase the overall ROS content in monocytes as demonstrated by flow cytometry using monocytes stained with CM-H_2_DCFDA (**Figure 2E**). Additionally, MYX1 did not have any effect on overall ROS content, in both unstimulated and stimulated cells. However, MYX2 treatment raised the ROS content irrespective of LPS stimulation (p=0.0313 and p=0.0269). Since MYX reduced ΔOCRV as measured by the Clark-type electrode (**Figure 2B**), we expected an overall decrease in ROS content rather than an increase. We therefore treated the monocytes with VAS2870. Inhibition of monocyte NOX with VAS2870 further raised ROS content upon MYX2 treatment (p=0.0357). In order to evaluate the impact of metabolic restriction on common activation markers, we analyzed the expression of CD14, CD16, CD80, HLA-DR, and CD11b on human monocytes after treatment with MYX. (**Figure 3**). At all levels of mitochondrial inhibition investigated, CD14 expression was reduced following treatment with LPS (data not shown). In the stimulated populations, MYX2 increased the proportion of CD16 positive cells (p=0.0469). Stimulation with LPS in the DMSO group reduced the CD16 positive population (p=0.0469). MYX mostly affected the expression of HLA-DR and CD11b. Following stimulation with LPS, there was an induction of HLA-DR in the group without MYX treatment (statistical trend, p=0.0781). However, in the stimulated group, MYX1 reduced the HLA-DR positive population as well as the median fluorescence intensity (MFI) (both p=0.0156), while MYX2 had no further effect compared to MYX1 treatment. The expression of CD11b was upregulated following the stimulation with LPS in the control cells (p=0.0156) and upon MYX1-treatment (p=0.0313). However, there was no increase following MYX2 treatment, and MYX1 reduced the expression in the stimulated group (p=0.0156).

### Metabolic Restriction Modifies Kinetics of Inflammatory Cytokines, But Has No Effect on Phagocytosis

**Figure 4:**
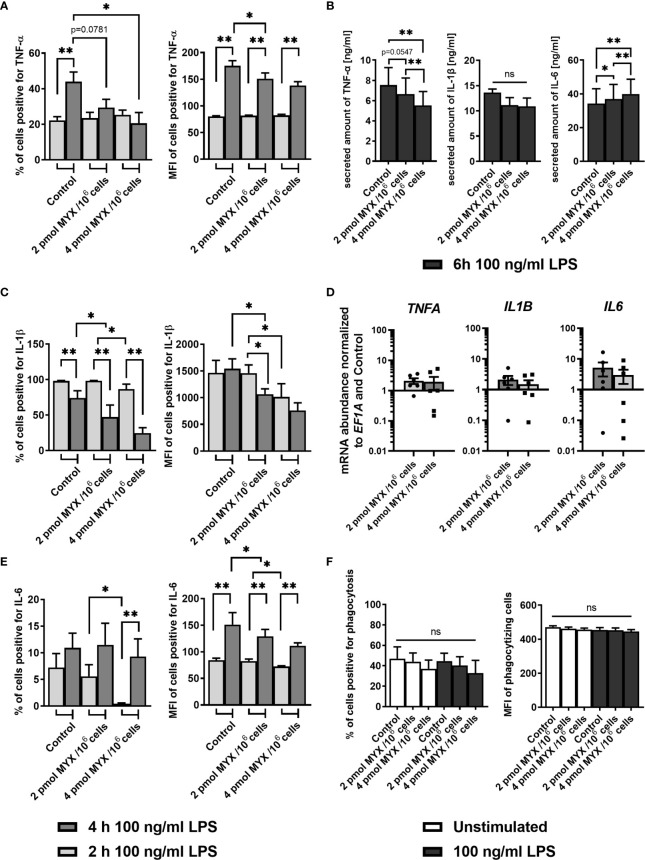
Metabolic restriction with MYX modifies the kinetics of the production of inflammatory cytokines but has no effect on phagocytosis. **(A–C)** Percentage and median fluorescence intensity of quiescent and stimulated (100 ng/mL LPS) human monocytes positive for intracellular staining of **(A)** TNF-α, **(B)** IL-1β and **(C)** IL-6 in the presence or absence of MYX (pmol/10^6^ cells) or vehicle control (1% (v/v) DMSO) under glucose free conditions. After 1 h of pre-stimulation with or without LPS, cells were treated with Brefeldin A (10 µg/mL) and stained following further 1 or 3 h of incubation (mean ± SEM; n = 6; *p < 0.05; **p < 0.01; Wilcoxon signed rank test and Mann Whitney U test). **(D, E)** Monocytes were cultured in glucose-free conditions stimulated for 6 h with both 100 ng/mL LPS and 2 pmol MYX/10^6^ cells (MYX1) or 4 pmol MYX/10^6^ cells (MYX2) or LPS alone. **(D)** Secreted amounts of TNF-α, IL-1β, and IL-6 were determined using ELISA (mean ± SEM; n = 8; *p < 0.05; **p < 0.01; Wilcoxon signed rank test). **(E)** Gene expression of *TNFA*, *IL1B* and *IL6* was normalized to the expression of *EF1A* and to the respective control (mean ± SEM; n = 6; *p < 0.05; **p < 0.01; Wilcoxon signed rank test). **(F)** Percentage and median fluorescence intensity (MFI) of monocytes positive for FITC-labeled *E. coli* bacteria after 1 h incubation in the presence or absence of LPS (100 ng/mL) and MYX (pmol/10^6^ cells) or 1% (v/v) DMSO (vehicle control) under glucose free conditions (mean ± SEM; n = 6; Wilcoxon signed rank test). ns, not significant.

Next, we assessed the capacity of human monocytes to produce inflammatory cytokines under metabolically restricted conditions. To this end, cells were stained for TNF-α, IL-1β and IL-6 after 1 hour of pre-stimulation with LPS and subsequent 1 or 3 hours of blocking with BFA (**Figures 4A–C**). After 1 hour, cells stimulated with LPS had higher proportions of cells positive for all three cytokines compared to unstimulated cells and this was still true after 3 hours of incubation (data not shown). At the first time point of 2 hours, both the proportion and the MFI of positive cells for staining with IL-6 and IL-1β (both p= 0.0156 and p=0.0313 for % of positive and MFI respectively) was reduced upon MYX2-treatment. For IL-6, after 4 hours, there was no difference between stimulated groups for % of positive cells. Following MYX2-treatment, monocytes reached the same levels as stimulated control (**Figure 4C**). For IL-1β, after 4 hours, the difference between MYX1- and MYX2-treatment vanished: instead, the reduction of both proportion and MFI of positive cells now occurred between control and MYX1-treatment (both p=0.0156) (**Figure 4B**). Regarding TNF-α, the increase of positive cells from 2 to 4 hours of incubation was abrogated for MYX1- and MYX2-treatment. The increase of the MFI, however, was significant for DMSO and following MYX-treatment, even though the maximum reached upon MYX-treatment was lower than the control (p=0.0313) (**Figure 4A**). Analyzing secreted amounts of TNF-α, IL-1β and IL-6 after 6 hours of incubation, we observed (i) a significantly gradual decline in secreted amounts of TNF-α, (ii) a numerical (not significant) decline in secreted amounts of IL-1β and (iii) a significantly gradual increase in secreted amounts of IL-6 (**Figure 4D**). Interestingly, the observed changes in cytokine production and secretion were not accompanied by a significant increase/decrease of TNF-α, IL-1β and IL-6 gene expression indicating a rapid translational instead of a sustained transcriptional adaption process (**Figure 4E**).

Since phagocytosis is a key function of human monocytes, we analyzed the effect of MYX-treatment on the phagocytosis of *E. coli* with or without LPS as a co-stimulation. MYX had no significant effect on the phagocytosis function of human monocytes (**Figure 4F**).

### Metabolic Restriction Increased the Capacity and Dependency of Monocytes for the Use of Fatty Acids But Reduced the Use of Glycolysis

**Figure 5:**
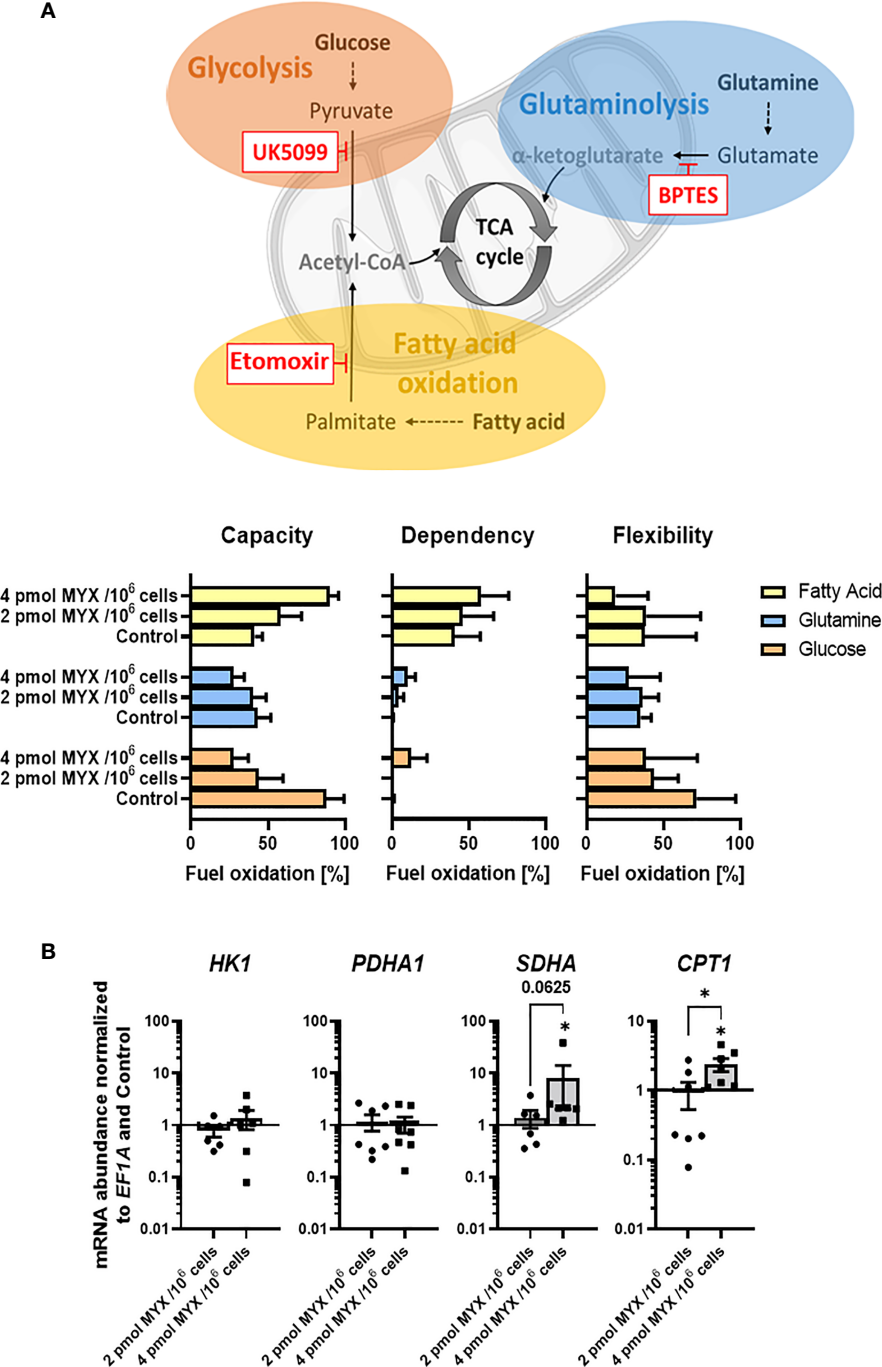
Metabolic restriction increased the capacity and dependency of monocytes for the use of fatty acids but reduced the use of glycolysis. **(A)** Seahorse XF Mito Fuel Flex Test was performed using 2 µM UK5099, 3 µM BPTES, and 4 µM etomoxir to block glucose oxidation, glutamine oxidation, and fatty acid oxidation, respectively (n = 3). Mitochondrial stress and glycolytic parameters were measured *via* OCR in pmol/min/1x10^5^ cells and are shown as percentage of max values and depicted as bars (mean ± SEM). **(B)** Gene expression of *HK1*, *PDHA1*, *SDHA*, and *CPT1* was normalized to the expression of *EF1A* and to the respective Control (mean ± SEM; n = 6; *p < 0.05; Wilcoxon signed rank test).

Next, we analyzed if metabolic restriction of LPS stimulated human monocytes impacts their capacity, dependency and flexibility to use certain cellular fuels namely glucose, glutamine and fatty acids in order to adapt and maintain cellular functions such as IL-6 production and phagocytosis (**Figure 5**). After initial LPS stimulation (100 ng/ml for 1 hour) and 4 hours of incubation, we analyzed if the cells’ mitochondria were able to compensate for the metabolic restriction by oxidizing other fuels (**Figure 5A**). Therefore, we added UK5009, BPTES, and etomoxir to block glucose oxidation, glutamine oxidation, and fatty acid oxidation (FAO), respectively. As expected, when gradually increasing the OXPHOS inhibition in the absence of glucose monocyte mitochondria reduced their capacity and flexibility to use glucose but also the use of glutamine. Interestingly, we observed that monocyte mitochondria were capable of compensating for the inhibition of glycolysis by increasing their capacity and dependency on FAO but not on glutaminolysis. To analyze if monocytes adapt to metabolic restrictions by transcriptional induction of key enzymes for the (i) first step of glycolysis (hexokinase 1; *HK1*), (ii) link of the glycolytic pathway and the tricarboxylic cycle (TCA) (pyruvate dehydrogenase complex E1 subunit α; *PDHA1*), (iii) connection of TCA and OXPHOS (Succinate dehydrogenase complex, subunit A of complex II of the electron transport chain; *SDHA*) and (iv) link of fatty acid oxidation to TCA and the generation of acetyl-CoA (carnitine palmitoyltransferase I; CPT1). As a result, we observed a significant increase of gene expression for *SDHA* and the mitochondrial long chain fatty acid transporter *CPT1* at MYX2 while *HK1* and *PDHA1* remained unaffected by metabolic restriction (**Figure 5B**).

## Discussion

In this study, we established a model of metabolically restricted conditions to mimic “battlefields” of human monocytes such as at sites of inflammation. A hallmark of these “battlefields” is the low availability of substrates such as oxygen and glucose (13). Additionally, low pH and increased levels of metabolites such as lactate render less effective synthesis of ATP and biosynthetic substrates, which are essential for cell activation (12, 14, 15). To reproduce those pathophysiological conditions *in vitro*, we cultivated the cells in medium without glucose and gradually reduced mitochondrial ATP production by inhibiting OXPHOS using myxothiazol (21). Myxothiazol is a specific inhibitor of respiratory chain complex III (CIII) that binds to the ubiquinol oxidation site Qo of CIII and blocks electron transfer from ubiquinol to cytochrome b, inhibiting CIII activity (21–24). Due to the competitive nature of this molecule, dimming the effect of OXPHOS inhibition without altering survival or mitochondrial ROS production provides the experimental prerequisite to analyze the metabolic effects after gradual inhibition of OXPHOS. Under these conditions, the pentose phosphate pathway, the serine synthesis pathway and the glycerol phosphate shuttle are heavily compromised since they critically depend on glucose carbon. This represents an important and pathophysiologically relevant restriction, since these pathways are known to be most important for metabolically active cells (13).

We first provide evidence that under such conditions the viability of the cells is not critically reduced. Next, we demonstrate MYX to effectively reduce the basal OCR of monocytes in the absence of glucose (**Figures 1**, **2**). The inhibitory effect of MYX on ΔOCRO was significant, even after a period of 6 hours. This indicates that the cells were on different and stable metabolic restriction levels in all functional experiments carried out during this period. It is known that monocytes can compensate for loss of glycolysis as a source of ATP production under conditions of glucose deprivation by an increase of the AMP/ATP ratio and enhanced FAO (25). Therefore, we measured the steady-state ATP content of cells, in order to confirm the different energy levels of the cells. Cells stimulated with LPS displayed lower ATP content than the unstimulated control, while the ΔOCRO was significantly higher in stimulated cells (**Figure 2**). This is likely because stimulation with LPS causes either higher ATP consumption by the cells or higher flux of carbon bodies into metabolic pathways to synthesize intermediates critical for cell activation or both, without a corresponding higher energy generation (25). Obviously, the decrease of ATP available could not be compensated by increased mitochondrial ATP production or by an increase in the AMP/ATP ratio resulting in an increased glycolytic energy supply mediated by AMP-activated protein kinase (AMPK) (26). The latter is explained by the fact that our model did not permit glycolysis. Instead, we demonstrate that monocytes were capable of compensating for the inhibition of glycolysis and gradual inhibition of OXPHOS by increasing their capacity and dependency on FAO but not on glutaminolysis confirming previous reports (25) (**Figure 5**). However, our study demonstrates the ability of monocytes to maintain the investigated immune functions to varying degrees even in the complete absence of glucose. Although glucose carbon is critical for metabolic pathways, which are involved in cell activation by providing intermediates, human LPS-stimulated monocytes (without MYX treatment) remain functional under conditions of glucose deprivation. The basal OCR and basal OCR for LPS-stimulated monocytes measured in our study is comparable to values found in the literature (25, 27). Additionally, NOX dependent OCR of the monocytes in our study was similar to that of Raulien and colleagues, corresponding to approximately one third of total OCR following LPS stimulation (25). This indicates that the relative production of ROS through NOX was probably similar in both studies. Regardless of NOX, there are two possible explanations for the comparable iROS levels in control and upon MYX1-treatment: First, either mechanisms protecting against ROS such as the glutathione peroxidase system were no longer sufficiently effective [biosynthesis of glutathione requiring ATP (28)], and/or NOX independent iROS were produced following MYX1 treatment (29). Indeed, MYX2 raised iROS higher than both MYX1 and untreated control cells regardless of LPS treatment, while blocking NOX by using VAS-2870 increased this effect significantly (**Figure 2**) indicating a NOX-independent mitochondrial ROS production (29, 30). Increased iROS, as observed in our study, has been demonstrated to interfere with the MAPK-p38 activation following LPS stimulation (31); a process which has recently been attributed to non-mitochondrial iROS (32). Second, the loss of effectiveness of protection mechanisms against iROS as induced by the metabolically compromised conditions increased NOX independent iROS. We see a possible explanation for this in the glutathione peroxidase system, whose biosynthesis is dependent on ATP and intermediates of the glycolysis feeding the pentose phosphate pathway (28). Furthermore, glutathione is a known off-target for the pan-NOX inhibitor VAS-2870 used in our study (33, 34), which also may explain the significant increase in iROS (**Figure 2**) following VAS-2870 treatment, even with blocked NOX as source of ROS.

Further, we demonstrate that phagocytosis and the LPS-induced production of IL-6 to remain functional even under extremely comprised metabolic conditions. Thus, IL-6 signaling and phagocytosis emerge to be of central importance to human monocytes where glycolysis, metabolic pathways that diverge from glycolysis, and OXPHOS are very much restricted or – in complete absence of glucose – even absent. In contrast, we show that the production of iROS and the expression of surface markers are dispensable at very restricted metabolic conditions as those were the first to become negatively affected. To the best of our knowledge, this is the first report emphasizing such hierarchy of monocyte functions under gradually metabolically restricted conditions mimicking “real-world” pathophysiological scenarios.

We found phagocytosis to be metabolically seen the most important immune process of human monocytes. Monocytes belong to the professional phagocytes, the group of cells with a broad range of particles that can be taken up (35). Our results concerning metabolic restriction and phagocytosis matches previous work by our lab, confirming the validity of our model (36). Previously, we revealed that phagocytosis by human monocytes under glucose-free conditions could only be influenced, by MYX quantities reducing the basal OCR to 20% compared to the untreated control (36). Therefore, it is not surprising that phagocytosis remained fully functional, since in this study the lowest basal OCR was 33% compared to the control (**Figure 4**). The expression of CD11b and HLA-DR markers are commonly used in the assessment of antigen presentation and transendothelial migration capacity of human monocytes (2, 37, 38) (**Figure 3**). MYX treatment reduced the expression of CD11b and HLA-DR in human monocytes. Both the low availability of ATP and the reduction of intermediates are likely to compromise the cell adhesion, signaling, migration and presentation of antigens, while phagocytosis remains fully functional (37, 38) (**Figure 3**).

A general feature of monocytes is the production of cytokines in response to a variety of stimuli, such as found in the inflamed joint of RA patients (39). Furthermore, it is well established that monocytes contribute to the pathogenesis of sepsis by secreting inflammatory cytokines such as TNF-α, which peaks in the first two hours and falls to undetectable levels after 6 hours. The resulting “cytokine storm” is thought to be of central importance in the pathogenesis of sepsis and seen as a potential therapeutic target (40). Upon stimulation with LPS, human monocytes show a near linear increase for cellular IL-1β, IL-6 and TNF- α during the first 6 hours of incubation, as shown by others (41). Therefore, mainly ATP and carbon body availability should determine differential production kinetics for these cytokines in our study. IL-1β production was the most affected by reduced ATP availability and lack of glucose supply although the amount secreted remained unchanged (**Figure 4**). In the case of TNF-α, shortage of ATP and lack of glucose had a similar effect on the percentage of TNF-α-positive cells over time. However, monocytes at all metabolic levels investigated could still increase cellular TNF-α levels over time, but to a lesser extent under high metabolic stress finally leading to a gradual decline of secreted amounts of TNF-α. Interestingly, the induction of IL-6 showed a strong compensatory reaction over time in response to shortage of ATP and glucose and increased in the levels secreted after 6 hours. Our data for IL-6 demonstrate that after 4 hours, monocytes can still produce the same amount of IL-6, regardless of ATP and glucose availability. Of note, secretion of TNFα and IL-1β rely on prior proteolytic steps whereas secretion of IL-6 does not, which might contribute to differences in the secretion profile of these cytokines (42, 43). Although these data are consistent with our observations using flow cytometry and are therefore likely to be directly related to the amounts of cytokines produced, we cannot completely exclude proteolytic steps of TNFα and IL-1β that were influenced by the applied metabolic stress. On the other hand, proinflammatory properties of IL-6 are mainly based on the IL-6 trans-signaling mechanism, in which IL-6 bound to the soluble IL-6R that can stimulate virtually all cells in the human body, whereas only a few cells also respond to IL-6 alone (44). Interestingly, secreted amounts of the IL-6/IL-6Rα complex in the medium of isolated monocytes cultured under glucose-free conditions for up to 6 hours were almost undetectable and not inducible by LPS or affected by metabolic stress (data not shown). Thus, we suggest that either another signal is necessary of or IL-6/IL-6Rα complex is in general induced when glycolysis is available. Finally, IL-6/IL-6Rα complex has facilitates a long-lasting IL-6 availability and activation of target cells which follows a different kinetic (44). Nevertheless, our results underline the importance of TNF-α and IL-6 in human monocytes. Since TNF-α is known to mediate the IL-6 production by human monocytes in the arthritic joint, both, anti-IL-6 receptor and anti-TNF-α targeted antibodies proved to be very effective in the treatment of RA (45, 46).

Our model sought to heavily interfere with the energy and intermediate household of human monocytes in order to test their compensation limits and to assess the metabolic-dependent effects on their functionality. Modeling restricted energy and intermediate supply and decreased mitochondrial reserve capacity in human monocytes with mitochondria as primary ATP source such as established in our study could provide useful information in pathophysiology of human monocyte mediated inflammatory diseases such as rheumatoid arthritis (RA) (47) and atherosclerosis (48). It should be noted that the prevalence of both diseases increases with age (49, 50), while ageing impairs mitochondrial reserve capacity of monocytes (51).

It should be noted finally, that we intentionally chose not to use differential rates of glycolysis in our model. Establishing a model with a similar goal to ours, but including gradual inhibition of glycolysis, would have interfered with the transcription process of TNF-α mRNA (52) and CD11b expression (32). The informative value of those outcomes would have been reduced. Moreover, the role of the glycolytic pathway in human monocytes/macrophages remains a subject of debate. Several recent studies using different metabolic assessment methods depict monocytes/macrophages as rather glycolytic cells. However, other recent studies contradict these results by using different methods and/or studying human instead of murine cells (11, 53–57). The limitative value of an absent glycolysis might therefore remain of minor extent for the scope of our study.

## Conclusion

Our results demonstrate a clear hierarchy of the LPS-induced immune processes in human monocytes as induced by metabolically restricted conditions mimicking pathophysiological conditions. Of the assessed processes, phagocytosis and the production of IL-6 were the highest in the hierarchy, followed by ROS production through NOX, expression of surface markers and the production of TNF-α and IL-1β. By applying our method to cells of patients with diseases under conditions of metabolic restrictions, future projects could identify most critical and, therefore, most promising therapeutic targets, and could obtain a better and more translational understanding of monocyte-mediated diseases.

## Data Availability Statement

The raw data supporting the conclusions of this article will be made available by the authors, without undue reservation.

## Ethics Statement

The studies involving human participants were reviewed and approved by EA1/207/17 Charité—Universitätsmedizin Berlin. The patients/participants provided their written informed consent to participate in this study.

## Author Contributions

P-LK, MP, TG, and FB designed the study. P-LK, MP, AD, TB, YC, TG, and FB collected, analyzed and interpreted the experimental data on the *in vitro* model. P-LK, MP, TB, TG, and FB prepared the main manuscript text. All authors contributed to writing or reviewing the manuscript and final approval.

## Funding

This work was supported by the Charité internal research funding. The work of TG was funded by the Deutsche Forschungsgemeinschaft (project no. 353142848). AD was supported by the Studienstiftung des deutschen Volkes and by the Joachim Herz Foundation (Add-on Fellowship 2020).

## Conflict of Interest

The authors declare that the research was conducted in the absence of any commercial or financial relationships that could be construed as a potential conflict of interest.

## Publisher’s Note

All claims expressed in this article are solely those of the authors and do not necessarily represent those of their affiliated organizations, or those of the publisher, the editors and the reviewers. Any product that may be evaluated in this article, or claim that may be made by its manufacturer, is not guaranteed or endorsed by the publisher.
