# Supplementary material for: Production of IL-6 and Phagocytosis Are the Most Resilient Immune Functions in Metabolically Compromised Human Monocytes
Source: Front Immunol. 2021 Oct 19;12:730672. doi: 10.3389/fimmu.2021.730672 (PMC8562108; doi:10.3389/fimmu.2021.730672)
Supplement: Supplementary file 1 [file DataSheet_1.pdf]

Supplementary Material

1 Supplementary Figures

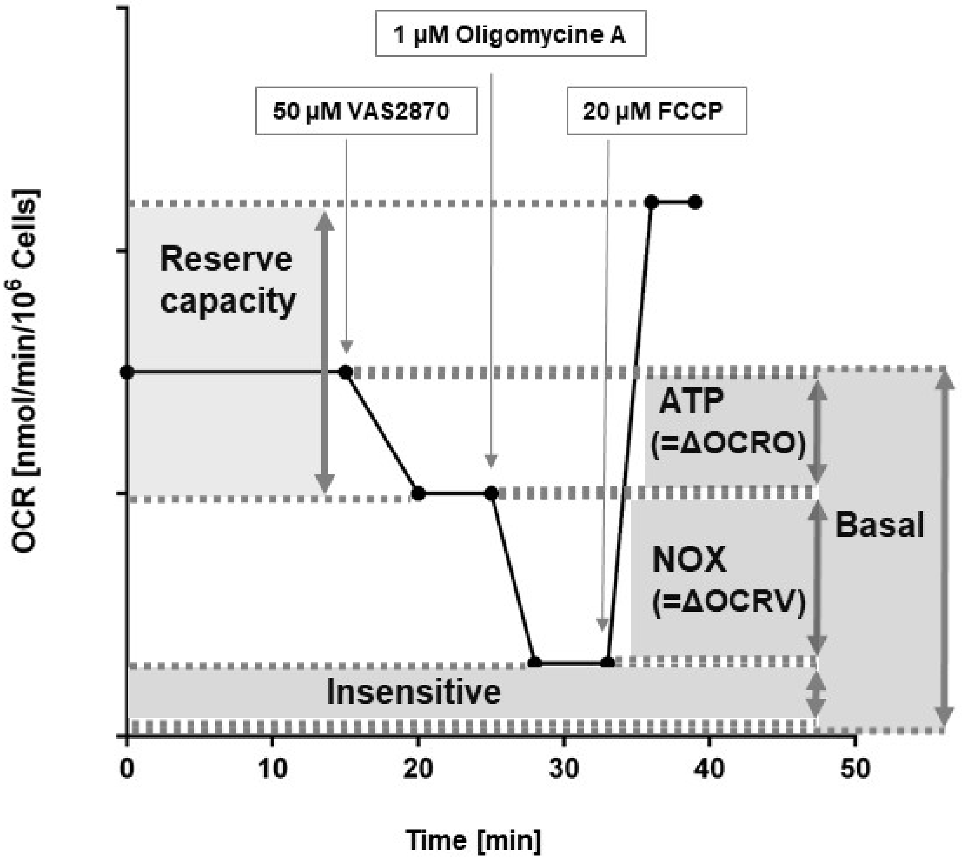

Supplementary Figure 1. Time protocol of OCR measurement of human monocytes after 2 and 6h incubation. 50 µM VAS2870 at t=15 min 1 µM Oligomycin A at t= 25 min, 20 µM FCCP at t= 33 min.

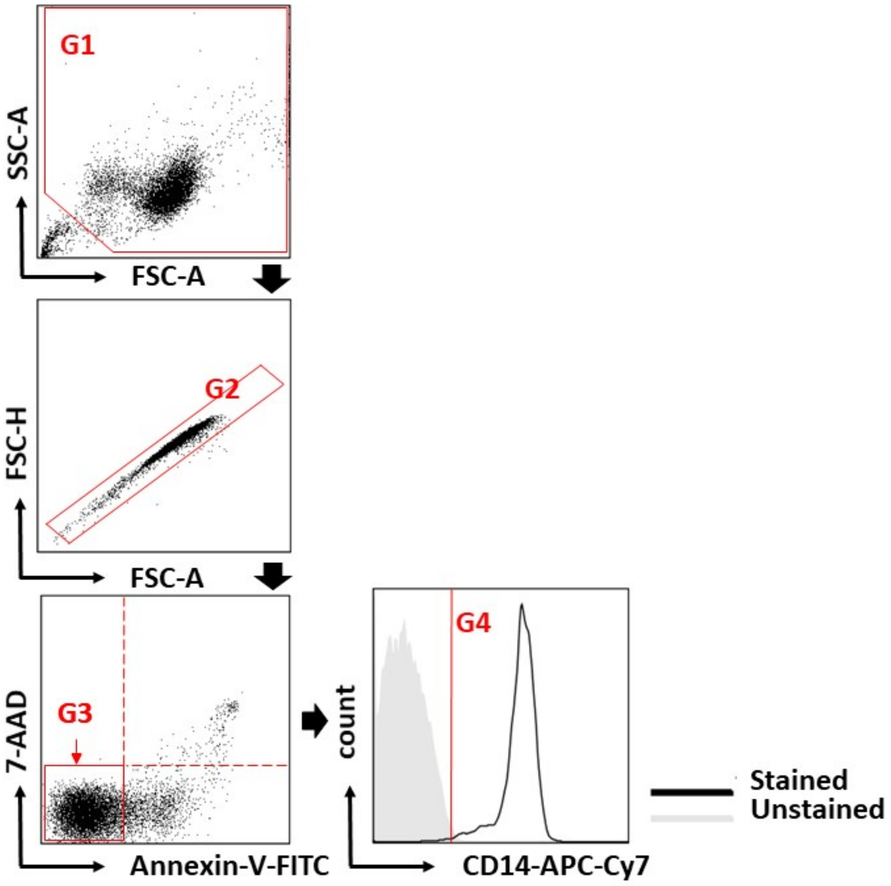

Supplementary Figure 2. Gating strategy: Assessment of viability and purity following isolation and /or incubation by flow cytometry. Detritus (FSC/SSC, G1) and cell doublets (FSC-A/FSC-H, G2) were excluded, followed by gating for viability (7-AAD/Annexin-V, G3) and purity (count/CD14, G4).

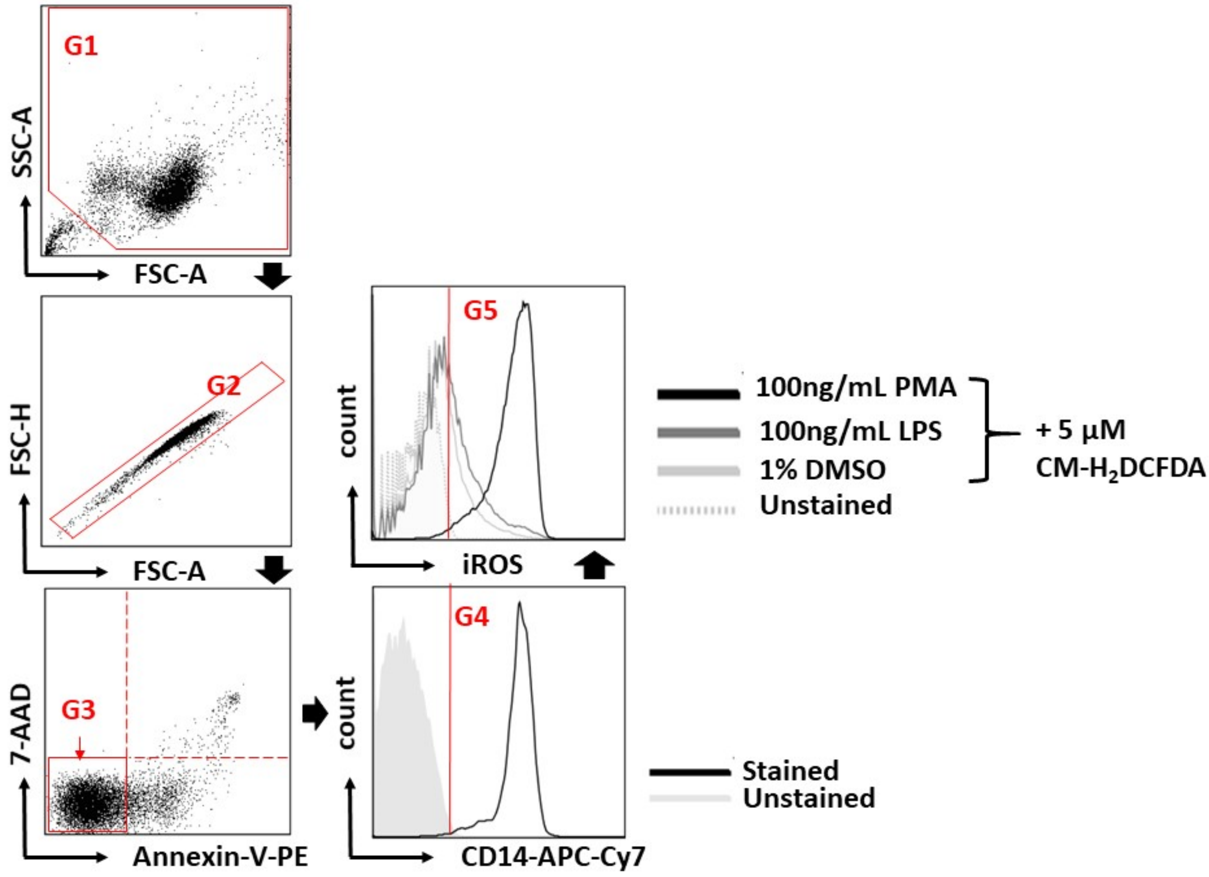

Supplementary Figure 3. Gating strategy: Assessment of intracellular reactive oxygen species by flow cytometry. Detritus (FSC/SSC, G1) and cell doublets (FSC-A/FSC-H, G2) were excluded, followed by gating for viable (7-AAD/Annexin-V, G3) and CD14-positive cells (count/CD14, G4). Median fluorescence intensity of cells positive for CM-H_2DCFDA staining was assessed by G5.

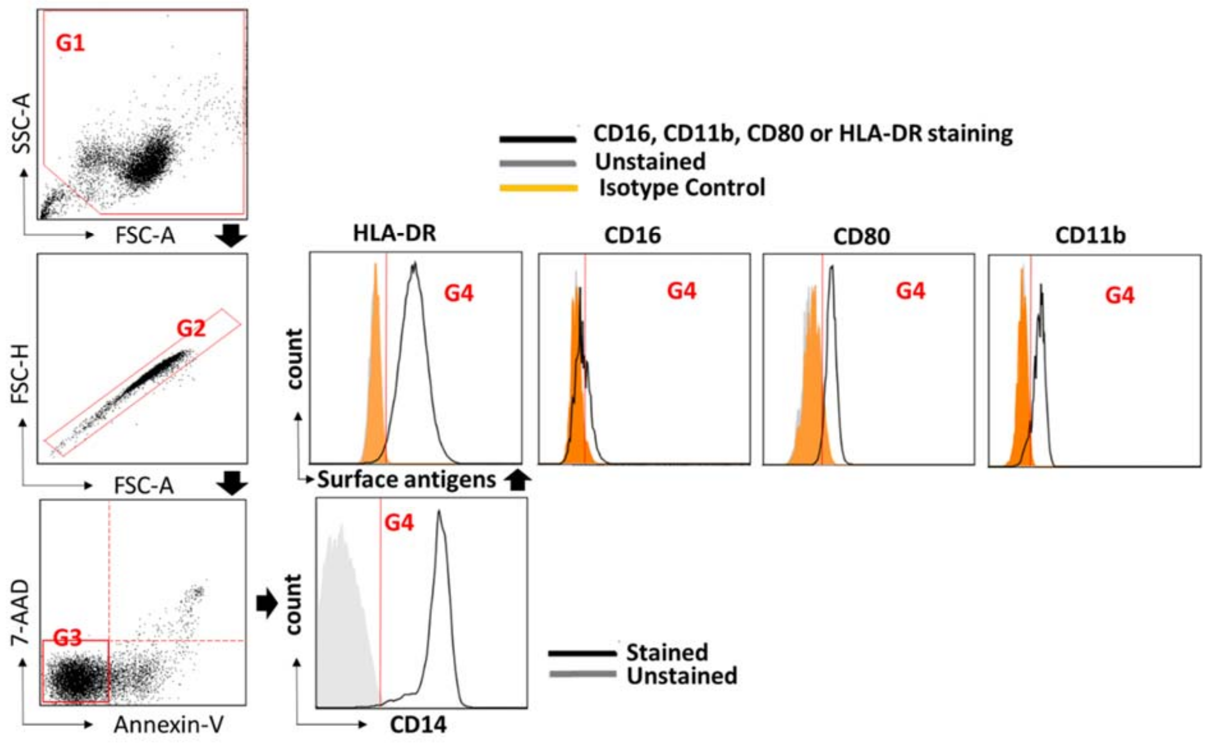

Supplementary Figure 4. Gating strategy: Assessment of surface markers of activation by flow cytometry. Detritus (FSC/SSC, G1) and cell doublets (FSC-A/FSC-H, G2) were excluded, followed by gating for viable (7-AAD/Annexin-V, G3) and CD14-positive cells (count/CD14, G4). Median fluorescence intensity (in comparison to isotype control staining) and percentage of cells positive for surface antigen staining was assessed by G5.

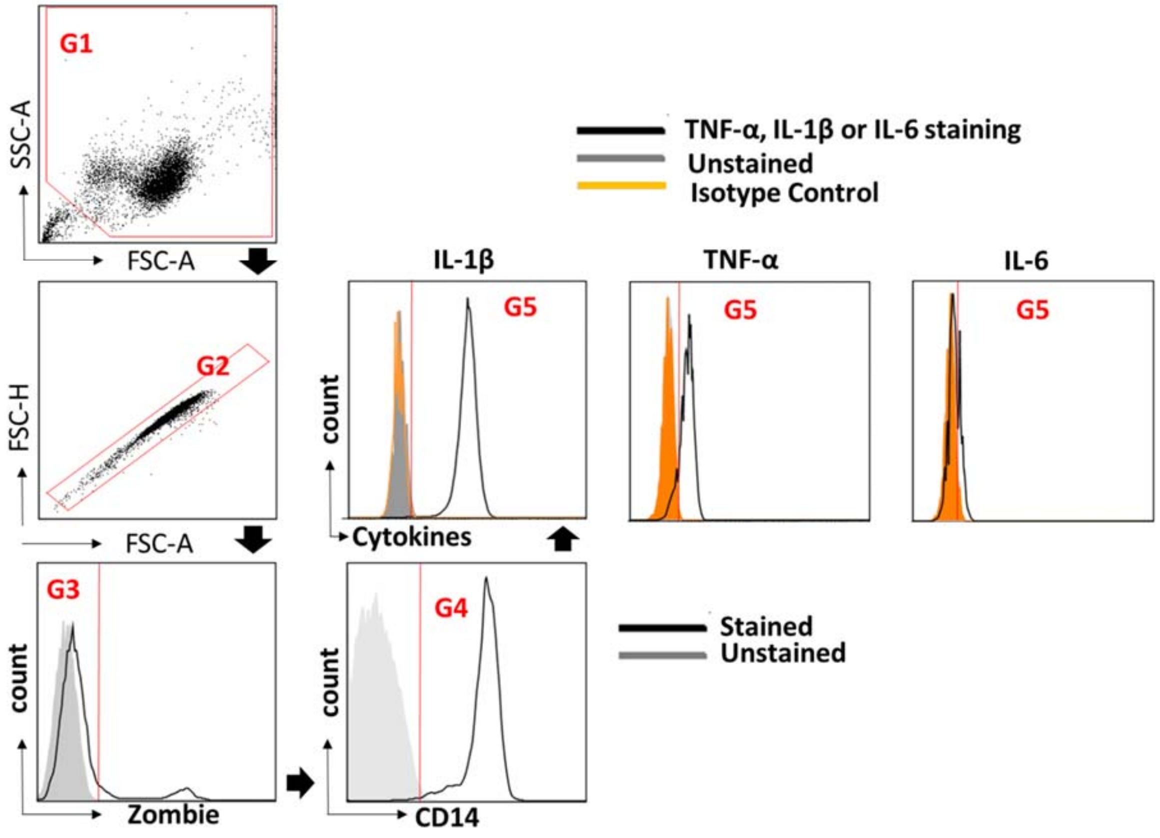

Supplementary Figure 5. Gating strategy: Assessment of intracellular cytokines by flow cytometry. Detritus (FSC/SSC, G1) and cell doublets (FSC-A/FSC-H, G2) were excluded, followed by gating for viable (count/Zombie, G3) and CD14-positive cells (count/CD14, G4). Median fluorescence intensity (in comparison to isotype control staining) and percentage of cells positive for intracellular cytokine staining was assessed by G5.
